# The “Beacon” Structural Model of Protein Folding: Application for Trp-Cage in Water

**DOI:** 10.3390/molecules28135164

**Published:** 2023-07-02

**Authors:** Qiang Sun, Xian He, Yanfang Fu

**Affiliations:** Key Laboratory of Orogenic Belts and Crustal Evolution, Ministry of Education, The School of Earth and Space Sciences, Peking University, Beijing 100871, China; xianhe@stu.pku.edu.cn (X.H.); yanfang.fu@stu.pku.edu.cn (Y.F.)

**Keywords:** protein folding, water, hydrophobic interactions, hydrogen bonding, necessary pathway

## Abstract

Protein folding is a process in which a polypeptide must undergo folding process to obtain its three-dimensional structure. Thermodynamically, it is a process of enthalpy to overcome the loss of conformational entropy in folding. Folding is primarily related to hydrophobic interactions and intramolecular hydrogen bondings. During folding, hydrophobic interactions are regarded to be the driving forces, especially in the initial structural collapse of a protein. Additionally, folding is guided by the strong interactions within proteins, such as intramolecular hydrogen bondings related to the α-helices and β-sheets of proteins. Therefore, a protein is divided into the folding key (FK) regions related to intramolecular hydrogen bondings and the non-folding key (non-FK) regions. Various conformations are expected for FK and non-FK regions. Different from non-FK regions, it is necessary for FK regions to form the specific conformations in folding, which are regarded as the necessary folding pathways (or “beacons”). Additionally, sequential folding is expected for the FK regions, and the intermediate state is found during folding. They are reflected on the local basins in the free energy landscape (FEL) of folding. To demonstrate the structural model, molecular dynamics (MD) simulations are conducted on the folding pathway of the TRP-cage in water.

## 1. Introduction

Proteins play essential roles in biological processes, functioning as exquisite catalysts, inhibitors, and sensors. To perform the functions, most proteins are necessarily folded into compact three-dimensional structures, native states, and remain stably folded. To predict the native structure of a protein from its primary structure, it is important to understand the process of protein folding. Thus far, many experimental and theoretical works have been devoted to investigating the mechanism of protein folding.

Protein folding is a process of molecular self-assembly, during which a disordered polypeptide chain will collapse to form a compact and well-defined three-dimensional structure. It is one of the most important problems of molecular biology. From an Anfinsen experiment on ribonuclease A, this states that the information required to form folded conformation resides in its polypeptide amino acid sequence as the denatured enzyme refolds into native conformation without assistance of any other protein [1]. According to the Levinthal study [2], it means that protein folding cannot take place via a random sampling of all possible conformations. Additionally, many physical models have been proposed to explain the high speed of protein folding, such as the nucleation growth model [3,4], framework model [5,6], diffusion–collision mechanism model [7,8], hydrophobic collapse model [9,10], and jigsaw model [11]. In these “classical views” of protein folding, it was assumed that proteins could find their predestined native states within the vast space of possible folds only by traveling through some predetermined pathway (or intermediates), which are considered to be partially unfolded conformations that are stable enough to be detected.

In recent years, the free-energy folding funnel model, derived from hypothetical energy landscape and statistical mechanical considerations [12,13,14,15,16,17,18], was developed to investigate protein folding. In this model, proteins are in disordered states at the highest energy level. As the proteins fold into the organized, native-like conformation, they shift to a lower energy phase. The energy landscape theory states that a folding of a protein does not follow a singular, specific pathway but occurs through statistical description of the topography of the free-energy landscape [19]. Therefore, it suggests that predefined pathways with compulsory intermediates simply do not exist. However, this is incompatible with the work of Englander, Mayne, and co-workers [20]. Based on hydrogen exchange studies, they suggested that a protein will fold by foldons if the protein contains foldons because this route is the fastest route for folding [21,22,23,24,25,26]. The foldon evidence indicates that protein folding follows a single folding pathway because the pathway is foldon-directed. To date, there is a strong debate on the folding pathways of proteins in water, with single or multiple pathways.

Hydrophobic interactions play an important role in protein folding. Therefore, it is important to study the physical origin of hydrophobic interactions. Historically, the classical mechanism for hydrophobicity, proposed by Frank and Evans [27] and advanced by Kauzmann [28] and many others, was based on the “iceberg” model. This means that the water immediately surrounding the hydrophobic group is more “ordered” than in bulk water as a small hydrophobic solute (such as argon or methane) is embedded into water. Later, it was recognized that hydrophobic interactions may be related to the solute size. When large hydrophobic solutes are inserted into water, as suggested by Stillinger [29], a distorted hydrogen bond network cannot be maintained in the water layer next to an extended hydrophobic surface, and the hydrogen bond network is then disrupted. In Lum, Chandler, and Weeks (LCW)’s [30,31,32] works, they provided a quantitative description of structural and thermodynamic aspects of hydrophobic hydration over the entire small to large length scale region. The crossover from small- to large-scale behavior occurs as the solute radius is about 1 nm. In our recent works [33,34,35,36,37,38,39], hydration-free energy is derived and used to understand the mechanism of hydrophobic effects. They are ascribed to the structural competition between interfacial and bulk water.

In combination with our recent studies [33,34,35,36,37,38,39] on hydrophobic interactions, this work is devoted to investigating the folding mechanisms of proteins in water. Thermodynamically, it is an enthalpic process to overcome the loss of conformational entropy in folding. Protein folding is primarily related to hydrophobic interactions and intramolecular hydrogen bondings. Hydrophobic interactions play an important role in the whole folding process, especially in the initial structural collapse of a protein. Additionally, based on thermodynamic analysis, folding is reasonably guided by the strong interactions within proteins, such as intramolecular hydrogen bondings related to the α-helices and β-sheets of the secondary structures. Therefore, proteins are divided into FK regions related to intramolecular hydrogen bondings and non-FK regions. During folding, various conformations are expected for FK and non-FK regions, such as specific and dynamic conformations. Different from non-FK regions, it is necessary for FK regions to form specific conformations during folding, which are regarded as the necessary pathways (“beacons”) in folding. Additionally, they are reflected on the local basins in the FEL of folding. To demonstrate the structural model, MD simulations are conducted on the folding pathway of the TRP-cage in water.

## 2. Hydrophobic Interactions

Protein folding is a physical process, during which a disordered polypeptide chain is collapsed and folded to attain a compact and well-defined three-dimensional structure. According to Anfinsen’s study [1], this means that the three-dimensional structure of a native protein may be the one in which the Gibbs free energy of a whole system is at its lowest. Therefore, under physiological conditions, a protein tends to be in its most stabilized form and remains biologically active in its three-dimensional structures. During folding from the extended to folded conformations, the lowest Gibbs energies may be expected for the folding pathways of proteins in water.

Protein folding may be related to various forces exerted on the atoms of the amino-acid chain, such as hydrophobic interactions, hydrogen bondings, van der Waals interactions, etc. These forces may be due to the interactions within a protein itself (direct forces), as well as related to the solvent (solvent-induced forces). Thermodynamically, as the solutes are embedded into water, the thermodynamic functions include water–water, solute–water, and solute–solute interaction energies,

(1) $$\Delta G=\Delta G_{Water-water}+\Delta G_{Solute-water}+\Delta G_{Solute-solute}$$

In fact, it is necessary for solutes to approach each other before they are affected by the direct solute-solute interactions (Δ*G_Solute-solute_*). This is closely related to Δ*G_Water-water_*, and Δ*G_Solure-water_*. Therefore, to understand the driving force during protein folding, it is necessary to investigate the structure of water, and the effects of solutes on water structure, respectively.

Numerous works have been carried out to understand the structure of water. To date, various structural models have been proposed, which are generally partitioned into the mixture and continuum models [40,41].

(2a) $$HB_{Mixture\;mod\;el}={\left(HB\right)}_{Low-density\;type}+{\left(HB\right)}_{High-density\;type}$$

(2b) $$HB_{Continuum\;mod\;el}={\left(HB\;networks\right)}_{Various\;bond-length\;and\;bond-angle}$$

In the mixture model, two distinct structural types are regarded to simultaneously exist in ambient water. For the continuum model, water comprises a random, three-dimensional hydrogen-bonded network. It may be characterized by a broad distribution of O-H⋯O hydrogen bond distances and angles. However, the structural networks cannot be “broken” (or separated) into distinct molecular species as in the mixture model. Thus far, liquid water is usually regarded as a tetrahedral fluid, which is based on the first coordination number $N_{c}=4\pi \rho \int_{r_{min}}^{r_{max}}r^{2}g_{OO}(r)dr$, where ρ means the density of water, and r_min_ and r_max_ are the lower and upper limits of integration in g_OO_(r). For ambient water, Nc is determined to be 4.3 [42] and 4.7 [43], respectively.

Water is generally regarded as an anomalous liquid, which is related to the hydrogen bondings of water. The OH vibrations are sensitive to hydrogen bondings of water and widely used to study the structure of water. From the Raman spectroscopic studies [44,45,46], as three-dimensional hydrogen bondings appear, various OH vibration frequencies correspond to different hydrogen-bonded networks in the first shell of a water molecule (local hydrogen bonding), and the effects of hydrogen bonding beyond the first shell on OH vibrations are weak. Therefore, when three-dimensional hydrogen bondings occur, different OH vibrations may be due to various local hydrogen-bonded networks of a water molecule.

At ambient conditions, the Raman OH stretching band of water may be fitted into five sub-bands, which are ascribed to OH vibrations engaged in various local hydrogen-bonded networks, such as DDAA (double donor–double acceptor, tetrahedral hydrogen bonding), DDA (double donor–single acceptor), DAA (single donor–double acceptor), DA (single donor–single acceptor), and free OH vibrations, respectively [44,45,46,47]. Therefore, a local statistical model (LSM) is proposed for ambient water, which means that a water molecule interacts with neighboring water molecules (in the first shell) through various local hydrogen bondings. Additionally, the hydrogen bondings of water may be influenced by the changes in temperature, pressure, dissolved salt, confined environment, etc.

When a solute is embedded into water, the solute–water interface occurs, which undoubtedly affects the structure of water. The OH vibrations are primarily dependent on the local hydrogen bondings of a water molecule. Therefore, the dissolved solute mainly affects the structure of interfacial water (the topmost water layer at the solute–water interface) [48]. In comparison with bulk water, no DDAA (tetrahedral) hydrogen bondings are found in interfacial water [48]. Therefore, the Gibbs-free energy of interfacial water, incurred by the solute, is expressed as

(3) $$\Delta G_{Solute-water}=n_{DDAA}\cdot \Delta G_{DDAA}\cdot R_{Interfacial\;\;water}$$

where *n_DDAA_* is the hydrogen bondings per water molecule of DDAA hydrogen bondings, ∆*G_DDAA_* means the Gibbs energy of DDAA hydrogen bondings, and *R_Interfacial water_* is the ratio of interfacial water to bulk water.

From the Raman spectroscopic studies [44,45,46], DDAA (tetrahedral) and DA are the predominant hydrogen-bonded networks in ambient water. Additionally, they are related to the structural changes across the solute–water interface. It is important to understand the characteristics of DDAA and DA hydrogen bondings. From our recent study [39], in comparison with a DDAA hydrogen-bonded network, the DA structural motif owns a lower enthalpy and a higher entropy and density.

**Figure 1:**
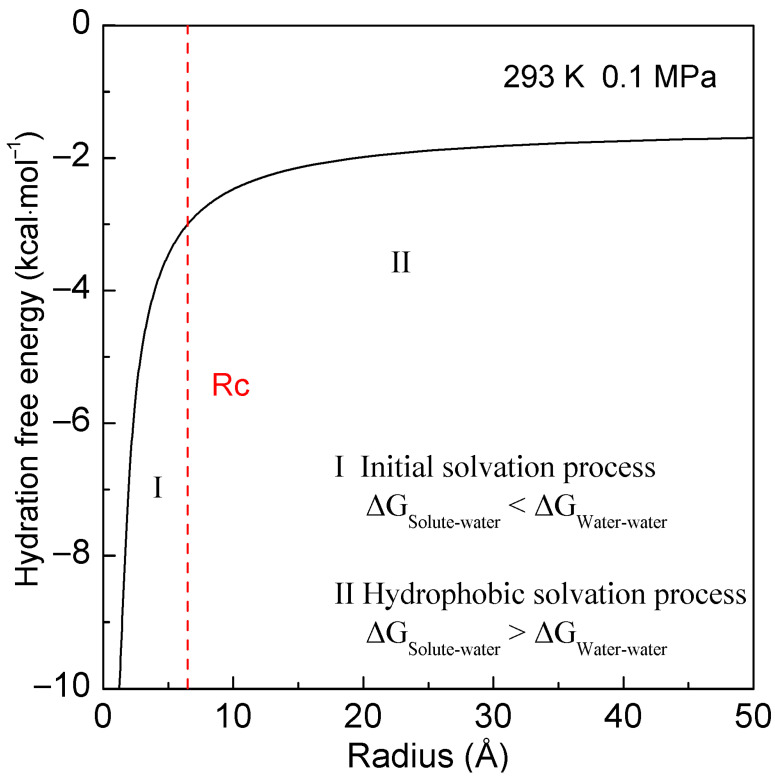
Hydration-free energy at 293 K and 0.1 MPa. With increasing the solute size, it is divided into the initial and hydrophobic solvation processes. The critical radius of solute (*Rc*) is shown by the dashed line.

Hydration-free energy means the change in Gibbs energy as a solute is transferred from a vacuum (or the gas phase) to a solvent. After the solute is simply regarded as a sphere, the *R_Interfacial water/volume_* is 4·*r_H_*_2*O*_/*R*, where *R* is the radius of solute. Therefore, hydration-free energy is given as (Figure 1)

(4) $$\begin{array}{ll} \Delta G_{Hydration} & =\Delta G_{Water-water}+\Delta G_{Solute-water}\\ & =\Delta G_{Water-water}+\frac{8\cdot \Delta G_{DDAA}\cdot r_{H2O}}{R} \end{array}$$

where Δ*G_Water-water_* is the Gibbs energy of water, and *r_H_*_2*O*_ is the average radius of a H_2_O molecule. At 293 K and 0.1 MPa, Δ*G_Water-water_* is −1500 cal/mol [49]. At ambient conditions, the average volume per water molecule is 3 × 10^−29^ m^3^. After it is treated as a sphere, *r_H_*_2*O*_ is determined to be 1.9 Å.

In thermodynamics, the lower the hydration-free energy the more stable the system is. Hydration-free energy is related to Δ*G_Water-water_* and Δ*G_Solute-water_*, and it may be dominated by Δ*G_Water-water_* or Δ*G_Solute-water_*. This means that the structural transition may take place as ∆*G_Water-water_* being equal to ∆*G_Solute-water_*,

(5) $$\Delta G_{Water-water}=\Delta G_{Solute-water}\;\;\;\;\;\;(Rc=\frac{8\cdot \Delta G_{DDAA}\cdot r_{H_{2}O}}{\Delta G_{Water-water}})$$

where *Rc* is the critical radius of the solute [33,39]. At 293 K and 0.1 MPa, *Rc* is 6.5 Å for a sphere solute [33]. With increasing the solute size (or concentrations), it is divided into initial and hydrophobic solvation processes (Figure 1). Additionally, Δ*G_Solute-water_* is proportional to the ratio of surface area to volume of solute (1/*R*), and various dissolved behaviors of solutes are expected in different solvation processes.

In the initial solvation process, Δ*G_Solute-water_* is less than Δ*G_Water-water_*, and hydration-free energy is dominated by Δ*G_Solute-water_*. To be more thermodynamically stable, this is fulfilled through maximizing Δ*G_Solute-water_*. In other words, it may be achieved through the maximization of the surface area to the volume ratio of the solutes in the water. Therefore, the dissolved solutes tend to be dispersed in solutions, and water molecules are found between them. Additionally, in the initial solvation process, the driving force is thermodynamically due to the increase in entropy arising from interfacial water.

**Figure 2:**
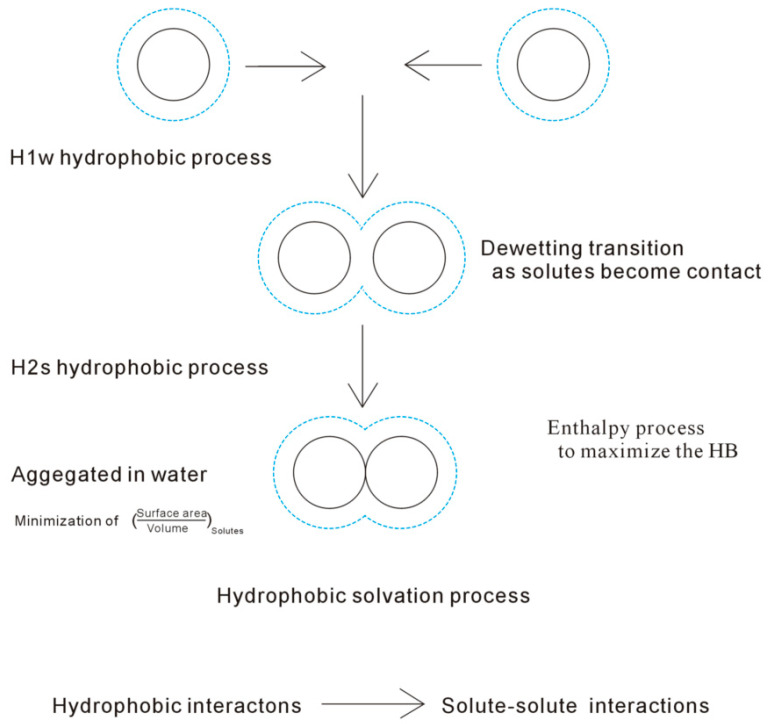
The mechanism of hydrophobic effects. The solutes mainly affect the structure of interfacial water, which is drawn by the dashed line. To maximize the hydrogen bondings of water, the dissolved solutes are attracted and tend to be aggregated in solution. During their association in water, they are divided into H1w and H2s processes. With decreasing the separation between solutes, the solute–solute interactions become important, especially in the H2s process.

In the hydrophobic solvation process, the Gibbs-free energy of interfacial water is higher than bulk water (Δ*G_Solute-water_* > Δ*G_Water-water_*). To be more thermodynamically stable, this may be fulfilled through maximizing Δ*G_Water-water_*. In fact, it is accompanied with the minimization of Δ*G_Solute-water_*. As Δ*G_Solute-water_* is proportional to the surface area to volume ratio of solute (1/*R*), the solutes may be aggregated to minimize the ratio of surface area to volume of them (Figure 2). Therefore, hydrophobic effects may be expected in the hydrophobic solvation process. In thermodynamics, the hydrophobic solvation process is ascribed to be an enthalpic process related to maximizing the hydrogen bondings of water.

The dissolved solutes mainly affect the hydrogen-bonded networks of interfacial water. Owing to hydrophobic interactions, they are attracted and aggregated in solutions to maximize the hydrogen bondings of water. In fact, as the solutes come into contact in solutions, this decreases the solute surfaces available for interfacial water. Therefore, during the solutes’ accumulations in water, the Gibbs energy of the interfacial water may be expressed as

(6) ΔGInterfacial water=γ⋅ΔGSolute−water (γ=(Surface areaVolume)Aggregate(Surface  areaVolume)Non−aggregate=f(1RSeparation))

where *γ* is the geometric factor, which is used to reflect the changes in solute surfaces while the solutes are aggregated in water. In fact, the solutes are rarely rigid. It may be accompanied with the changes in volume while they are accumulated in water. Therefore, *γ* is generally given as the above equation, where *R_Separation_* is the separation between the solutes. In our recent work [34], when the solute surfaces came into contact, the distances between them were termed as *R_H_* (hydrophobic radius).

While the solutes are accumulated in water, they are divided into H1w and H2s hydrophobic solvation processes [34]. In the H1w hydrophobic process, the distances between the solutes are larger than *R_H_*, or *γ* is 1 [34]. In other words, no accumulation of solute surfaces may be expected, and water molecules are found between the solutes. However, as the solutes come into contact in the H2s hydrophobic process, the distances between the solutes are less than *R_H_*, or *γ* is less than 1 (*γ* < 1). Additionally, while the solutes are aggregated in water, a dewetting transition process, similar to the liquid–gas phase transition, may be observed. From our recent study [34], dewetting is closely related to the H2s hydrophobic process, in which a single water layer between solutes may be expelled into bulk water, and the solute surfaces come into contact in solutions.

Additionally, based on our recent work [36], various directional natures are expected in the H1w and H2s processes. In the H1w hydrophobic process (>*R_H_*), the solutes tend to approach the specific direction with the lowest energy barrier, in which less water molecules are expelled. However, as the solutes come into contact in the H2s process (<*R_H_*), this decreases the solute surfaces available for interfacial water. The Gibbs-free energy of the interfacial water is proportional to the surface area to volume ratios of the solutes. Therefore, the solutes are expected to be aggregated in the specific direction to minimize the surface area to volume ratio. These may be used to understand the mechanism of molecular recognition, especially the specificity of molecular recognition.

In the hydrophobic solvation process, the solutes are expected to be aggregated to maximize the hydrogen bondings of water. While the solutes are associated in solutions, the interfacial water molecules in the region between the solutes may be expelled into bulk water, which may be closely related to the hydrophobic interactions. In other words, the strengths of the hydrophobic interactions may be dependent on the water molecular numbers transformed from the interfacial to bulk water. From this, it is expressed as

(7) $$\begin{array}{ll} \Delta G_{Hydrophobicity} & =\gamma \cdot \frac{8\cdot \Delta G_{DDAA}\cdot r_{H2O}}{R_{Solute-solute}}-\sum_{i=1}^{m}\frac{8\cdot \Delta G_{DDAA}\cdot_{H2O}}{R_{i}}\\ & =n_{Interfacial\to bulk\;water}\cdot \Delta G_{DDAA} \end{array}$$

where the first (second) item is Gibbs energy of interfacial water after (before) solutes are aggregated in water. Additionally, *n_Interfacial→bulk water_* is the water molecular number changed from interfacial to bulk water during the solutes’ associations in solutions. Based on Equation (7), hydrophobic interactions may be related to not only the solute size and shape but also temperature, pressure, and dissolved salt, etc.

In general, hydrophobic effects mean the tendency of non-polar molecules (or molecular surfaces) to be aggregated in water. According to our recent studies [33,39], they are reasonably described as the tendency for minimization of the ratio of surface area to the volume of the solutes to maximize the hydrogen bondings of water. This is because the dissolved solute mainly affects the structure of the interfacial water, and the hydrogen bondings of the interfacial water are weaker than the bulk water. Additionally, owing to the hydrophobic interactions, the solutes are attracted and tend to be aggregated in water. When decreasing the separation between solutes, the direct solute–solute interactions become stronger, especially when the solute surfaces come into contact in the H2s hydrophobic solvation process (Figure 2), which undoubtedly affects the dissolved behaviors of the solutes. Therefore, regarding the association of the solutes in water, it is ascribed to be driven by hydrophobic interactions.

## 3. Structure–Thermodynamics Relationship during Folding (“Beacon” Model)

In the process of protein folding, the interactions may include (1) hydrogen bondings, (2) hydrophobic interactions, (3) van der Waals interactions, (4) electrostatic interactions, etc. [50]. The process of protein folding is closely related to the forces exerted on the atoms of the amino acid chain, and the native folded structure is reasonably ascribed to the combined effects of the above interactions. In fact, these forces arise from the interactions with other parts of the protein itself (interactions within protein), as well as those related to water (water-induced forces). Therefore, various interactions may play a different role in the process of protein folding, which is related to the characteristics of each force.

In thermodynamics, Gibbs-free energy (Δ*G*) may be used to investigate whether a process is likely to occur, which is related to changes in enthalpy (Δ*H*) and entropy (Δ*S*),

(8) ΔG=ΔH−T⋅ΔS

where Δ*H* measures the total energy of a thermodynamic system, Δ*S* is a measure of the number of microscopic states of a system and is commonly used as a metric for disorder. When Δ*G* is less than zero, the process is spontaneous. Therefore, the process may be dominated by the changes in enthalpy (enthalpic process) or entropy (entropic process), respectively.

Thermodynamically, as a protein is folded into the native three-dimensional structure, the total Gibbs-free energy is reasonably expressed as follows,

(9) $$\begin{array}{ll} \Delta G & =\Delta G_{Water-water}+\Delta G_{Protein-water}+\Delta G_{Protein-protein}\\ & =\Delta G_{Hydration}+(\Delta H_{Protein-protein}-T\cdot \Delta S_{Protein-protein}) \end{array}$$

where ∆*G_Hydration_* is hydration-free energy, ∆*H_Protein-protein_* are the changes in enthalpy related to the interactions between the atoms (or residues) of protein, and ∆*S_Protein-protein_* is the conformational entropy, which means the loss of entropy during the protein folding from the extended chain to the compact native structure.

Protein stability refers to the energy difference between the folded and unfolded state of the protein in the solution, which determines whether a protein will be its native folded conformation or a denatured (unfolded or extended) state. Remarkably, the free energy difference between these states is usually between 20 and 60 kJ·mol^−1^ [51], which is of the magnitude of one to four hydrogen bondings. Therefore, folded proteins are only marginally stable [52]. This means that the unfolding–folding processes involve only the formations and break-ups of weak, non-covalent interactions. To investigate the mechanism of protein folding, it is important to correctly evaluate the dominant contributions among the many energy terms related to the free energy of protein folding.

The unfolded states of a protein possess an enormous number of degrees of freedom. During protein folding, accompanied with the decrease in conformational flexibility, this leads to an enormous loss of conformational entropy, which is a measure of the degrees of conformational freedom available to a protein (or part thereof). The loss of conformational entropy is a main destabilizing force in the thermodynamics of protein folding [53]. Experimental estimates of the entropy change on folding, and Δ*S_fold_* gives a comparable range of −2.6 to −9 cal∙mol^−1^∙K^−1^ per residue [54,55,56]. In fact, both the backbone and side-chain of each residue in a protein will have their freedom of motion restricted in the final folded structure. In Baxa et al.’s work [57], the loss of conformational entropy is largely due to the loss of backbone entropy. From Towse et al.’s study [58], the side chain entropy shows wider distributions on increasing side chain lengths or bulks.

Under physiological conditions, a polypeptide chain of a protein is spontaneously folded to a native three-dimensional structure. In thermodynamics, this means that the total Gibbs energy (Δ*G_Total_*), related to Δ*G_Hydration_* and Δ*G_Protein-protein_*, may be less than zero. In our recent study [39], hydrophobicity may have been closely related to the enthalpy-entropy compensation (EEC), which meant if Δ*H* and Δ*S* for the particular reaction were changing in one direction (either increase or decrease), their changes being transformed into Δ*G* were mutually compensated, and there was little change in the value of Δ*G*. Of course, this was due to the competition between the interfacial and bulk water [33]. To attain the native structure of a protein, it is essential to outweigh the loss of conformational entropy arising from folding. Therefore, it is necessary to obtain enthalpy enough to overcome the entropic penalty. This means that the protein folding is facilitated by maximizing the enthalpy, and it is reasonably described to be an enthalpic process.

There is considerable evidence that hydrophobic interactions must play a major role in protein folding [59]. According to Pace et al.’s study [60], the average contribution of hydrophobic interactions to a protein’s stability is 60%. The importance of methyl groups in modulating biological activity for small molecules is well documented. Experimentally, the benefit of burying a solvent-exposed methyl group on a ligand into a hydrophobic pocket of a protein is about 0.7 kcal∙mol^−1^, or a 3.2-fold increase in binding constant per methyl group (−CH_3_). In Pace et al.’s work [60], burying a −CH_2_ group on folding may contribute, on average, 1.1 ± 0.5 kcal∙mol^−1^ to protein stability. Additionally, based on Pace et al. studies [60,61] of 151 hydrogen bonding variants in 15 proteins, these mean that hydrogen bonding contributes to the protein stability about 40%, the net contribution of hydrogen bonding to overall protein stability is 1.1 kcal·mol^−1^, and is largely independent of the size of the protein [62]. Therefore, it can be derived that protein folding may be primarily affected by hydrophobic interactions and hydrogen bondings.

In general, as a protein folds, 81% of the nonpolar side chains, 70% of the peptide groups, 63% of the polar side chains, and 54% of the charged side chains are buried in the interior of the protein out of contact with water. Based on our recent studies [33,39], hydrophobic interactions play important roles in protein folding, especially in the initial (structural collapse) and final folding stages. To maximize the hydrogen bondings of water, a protein may be folded from the extended conformation into a three-dimensional structure. In kinetic experiments, an initial collapse in the size of the polypeptide chain is usually observed upon changing solvent conditions from being denaturing to renaturing [63,64,65,66].

Additionally, owing to hydrophobic interactions, this leads to the structural collapse of a protein in water. It may be transformed from an extended coil to a more compact, globular structure in order to minimize the surface area/volume ratio of protein. By decreasing the separations between the atoms (or residues) of proteins, the direct interactions between residues of proteins (interactions within protein) become stronger, which may affect the folding process of proteins in water. Therefore, it is found that protein folding may reasonably be regarded to be driven by hydrophobic interactions.

In thermodynamics, the loss of conformational entropy is a major destabilizing force in the process of protein folding. It has been considered that the entropic penalty can be compensated for by an energy gain through the formation of intramolecular hydrogen bonds in proteins [67,68]. Other intramolecular interactions, such as salt bridging, van der Waals attraction, etc., may also stabilize the native structures of proteins [69]. In fact, approximately two-thirds of the intramolecular hydrogen bonds are within repetitive elements of secondary structure [70] of the folded proteins. Based on the experimental studies [71,72], the hydrogen bond energy of the hydrogen bonds between the N-H groups and C=O groups of the main chains in the secondary structures is about −3.47 kcal∙mol^−1^. Therefore, the secondary structures of a protein, such as α-helices and β-sheets, are stabilized by the formation of intramolecular hydrogen bonds between the acceptor CO and the donor NH groups [73]. It is derived that the folding process may be guided by the formation of intramolecular hydrogen bondings within the secondary structures of protein. In fact, a backbone-based theory of protein folding is proposed [70,74], which is based on the energetics of backbone hydrogen bonds dominating the folding process. Of course, it is necessary that the hydrogen bonds between water and the peptide NH and CO groups must be broken before peptide hydrogen bonds are formed. This is undoubtedly related to the structural collapses of proteins driven by hydrophobic interactions.

Recently, high-resolution experimental methods sensitive to population distributions have been developed and applied to investigate the structural characteristics at different stages of folding reactions. Significant conformational heterogeneity is found during protein folding, including the unfolded state, collapsed intermediate states, and even the native state [75,76,77]. In fact, the heterogeneity in protein folding and unfolding reactions may be closely related to the various kinds of physicochemical interactions between various structural elements of a protein and between a protein and solvent [77].

From the discussion on the relationship between structure and thermodynamics during protein folding, a protein may be reasonably divided into the folding key (FK) and the remaining non-folding key (non-FK) regions, respectively. The Δ*G_Protein-protein_* is expressed as

(10) ΔGProtein−protein=ΔGFK Regions+ΔGnon−FK Regions+ΔGBetween FK and non−FK regions=∑i=1nΔGFKi+ΔGnon−FK Regions+ΔGBetween FK and non−FK regions

where Δ*G_FK regions_*, Δ*G_non-FK regions_*, and Δ*G_Between FK and non-FK regions_* mean the Gibbs energies within the FK regions, non-FK regions, and between the FK and non-FK regions, and *n* is the number of FK regions.

Intramolecular hydrogen bondings are expected to form within the FK regions of a protein, such as the α-helix and β-sheet of a secondary structure of a protein. Thermodynamically, protein folding is an enthalpic process. Therefore, folding may be guided by the strong intramolecular hydrogen bondings within the FK regions. Additionally, due to the formation of the intramolecular hydrogen bondings in the FK regions, a specific conformation may be expected for the FK regions in the folding process. This means that a higher structural order may be expected for the FK regions, which is related to the formation of intramolecular hydrogen bondings within the regions, in comparison with the non-FK regions. In addition, after the specific conformations are formed, they are usually preserved in the remaining folding time. In the process of protein folding, the FK regions may be regarded as the necessary pathways where folding tends to pass through so that the protein is folded to form the final three-dimensional native structure.

In comparison with the FK regions, other interactions are expected in the non-FK regions of a protein, such as van der Waals forces, salt bridges, hydrogen bondings between side chains of a protein, etc. They may play an important role in the final folding stage. Different from the specific conformation of FK regions, dynamic conformation is found for the non-FK regions in the folding process. In other words, evident conformational changes and fluctuations, such as in the distances, volumes, etc., may be expected for the non-FK regions of a protein during folding. Additionally, after the specific conformations of the FK regions are formed during folding, this may lead to decreases in the conformational changes and fluctuations related to the non-FK regions.

As a protein is folded from the extended chain to the native three-dimensional conformation in water, the protein tends to be engaged into the conformation with lower Gibbs energy to become more thermodynamically stable. In thermodynamics, it is facilitated by maximizing enthalpy to overcome the loss of entropy. During folding, this is related to not only folding time but also the spatial distributions of intramolecular interactions within a protein, especially FK regions. During folding, the Gibbs energy of Δ*G_protein-protein_* at time t may be reasonably expressed as

(11) $$\begin{array}{ll} \Delta G_{Protein-protein}\left(t\right) & =f\left(t,\sum_{i=1}^{m}x_{i}\right)=f\left(\sum_{i=1}^{m}x_{i}\left(t\right)\right)\\ & =f\left(FK\;region\left(t\right)\;,non-FK\;region\left(t\right)\right) \end{array}$$

where *m* is the atomic number of the protein, and *x_i_*(*t*) is the position of atom *i* in the protein. Therefore, at any folding time *t*, the protein is reasonably divided into the FK and non-FK regions.

**Figure 3:**
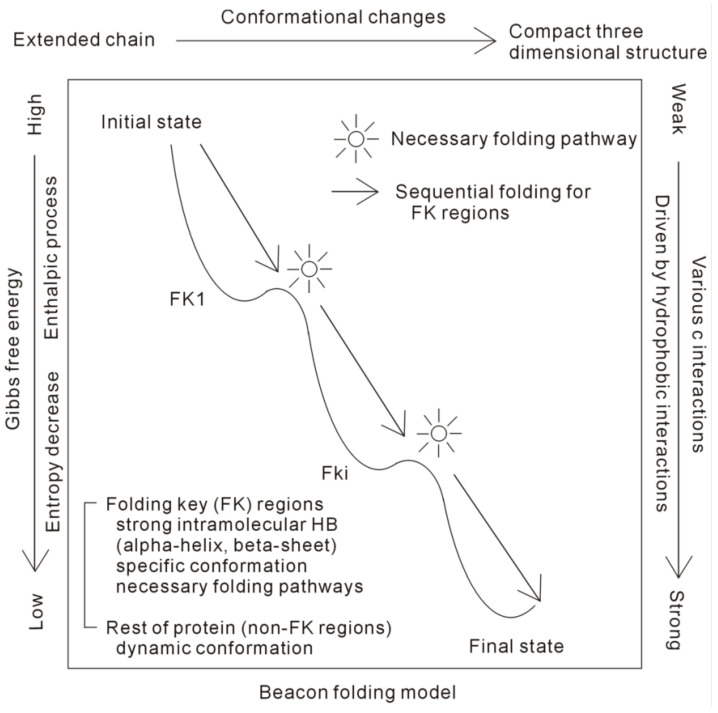
The “beacon” model of protein folding. The protein is divided into the FK and non-FK regions. During folding, the specific conformation is expected for FK, which is regarded as the “beacon” in protein folding. If there are many FK regions, the sequential folding pathway may be expected for them. Additionally, dynamic conformations are expected for the non-FK regions in the folding process.

From the above, folding may be divided into the following stages, such as the initial structural collapse, the folding of FKs related to intramolecular hydrogen bondings, and the last folding stage of the non-FKs. If several FKs exist, sequential formations (sequential folding) may be expected for them during folding, which are expressed as FK1, FK2, ⋯, etc. Additionally, the corresponding local basins are expected in the FEL in the folding pathway (Figure 3). In addition, after the specific conformations are formed within the FK regions, they tend to be preserved in the remaining folding time. Therefore, the sequential formations of specific conformations related to intramolecular hydrogen bondings, such as the α-helixes and β-sheets of secondary structures of a protein, are expected during folding.

Following the formation of the specific conformations related to the FK regions, it is engaged to the final folding stage. To attain the lowest Gibbs energy of a native three-dimensional structure during this stage, the structure may be modulated by various forces within the non-FK regions and those between the FK and non-FK regions, such as van der Waals forces, salt bridges, hydrogen bondings between side chains, and hydrophobic interactions. Generally, it is necessary for the protein to “breathe” so that water molecules in the interior may be repelled into the bulk. Of course, this is related to the dewetting transition of the H2s hydrophobic process.

Based on the experimental measurements [78,79,80], the molten globules (MG) [81,82] may be found in a specific region of a protein during folding. MGs are compact, partially folded conformations of proteins that have near-native compactness properties, substantial secondary structures, little detectable tertiary structures, and increased solvent-exposed hydrophobic surface areas relative to their native states, which are thought to be common intermediates in protein folding [83]. Later, there was a growing realization that the “dry” molten globule (DMG) [84] is another distinct state along a graduated MG spectrum. The defining difference between a DMG and a conventional MG is that the water has been squeezed from the core of a DMG [85]. From this work, a protein is divided into FK and non-FK regions in the folding process. Due to the intramolecular hydrogen bondings within the FK regions, higher structural orders may be expected for FK regions than the rest of the protein (non-FK regions) during folding. Therefore, this may be applied to understand the formation of MGs and DMGs in the folding process.

According to the thermodynamic analysis, a protein is reasonably divided into FK and non-FK regions. In the folding process, specific conformations may be expected for FK regions. These are different from non-FK regions, in which dynamic conformations may be found during folding. From these, various conformational changes are expected for FK and non-FK regions during folding. Therefore, it seems that there exist multiple folding pathways described as free-energy landscapes [19]. Additionally, the conformational changes may be related to not only the regions of protein (spatial distribution) but also the folding time (time dependence). In addition, folding is guided by the intramolecular hydrogen bondings within FK regions. During folding, sequential formations may be expected for the FK regions. In other words, evident conformational changes may be found at different folding times. It seems that there exists a single folding pathway for FK regions. In fact, these may be utilized to understand the strong debate on the folding pathways of a protein in water, either with single or multiple pathways.

**Figure 4:**
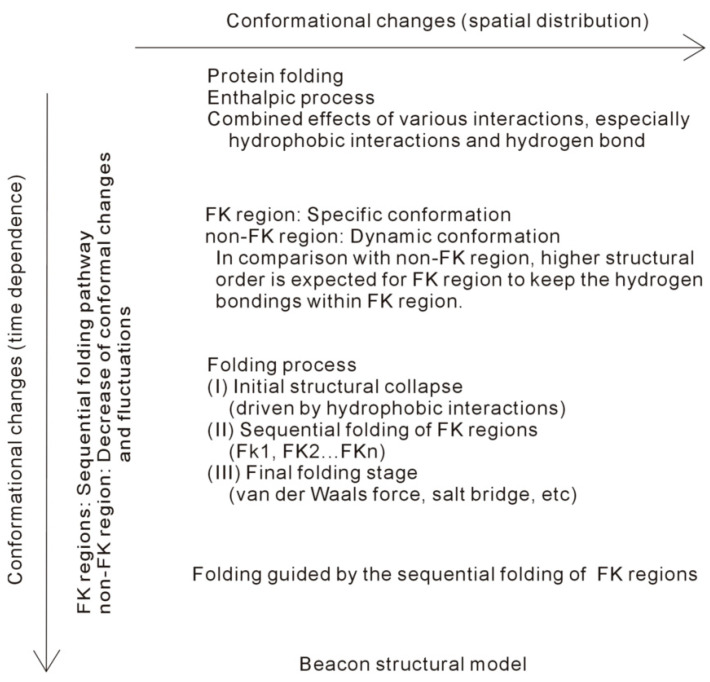
Conformational changes during protein folding.

In thermodynamics, protein folding is an enthalpic process, which is primarily related to hydrophobic interactions and intramolecular hydrogen bondings. In fact, hydrophobic interactions may play an important role in the whole process of protein folding, especially in the initial folding stage. A protein is divided into FK and non-FK regions, and various conformational changes are expected for them. Protein folding is guided by the strong intramolecular hydrogen bondings within FK regions, such as the secondary structures of a protein. In the folding process, sequential folding may be expected for the FK regions, which may be regarded as the “beacons”, where the folding might tend to pass through (Figure 4). In the final folding stage, to attain the lowest Gibbs energy of the native state, it is related to the combined effects of various forces related to the non-FK regions, such as van der Waals forces, hydrogen bondings between side chains, salt bridges, and hydrophobic interactions.

## 4. Application for Trp-Cage Folding

**Figure 5:**
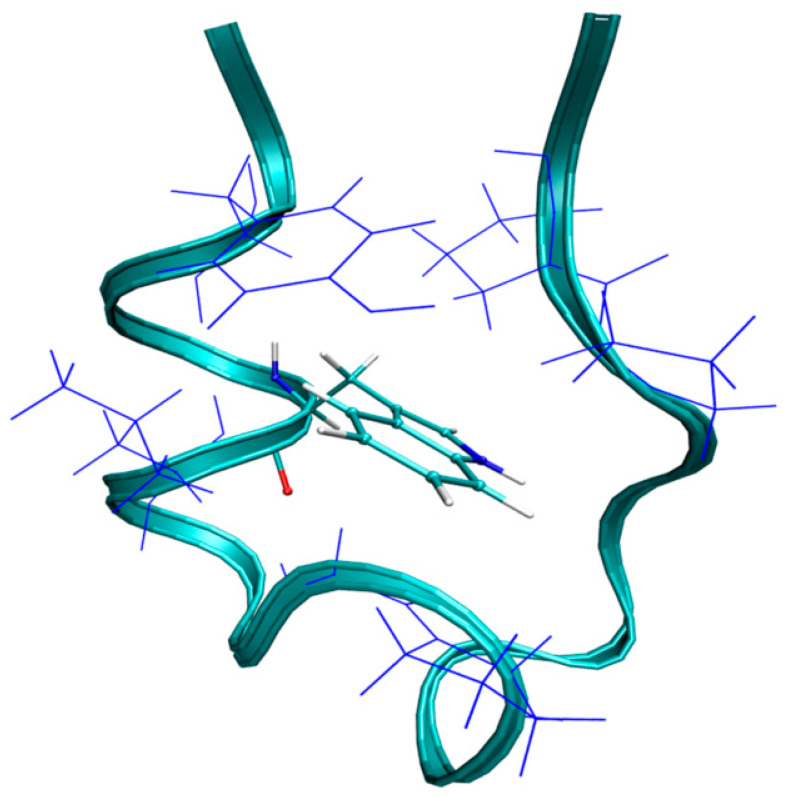
The structure of the TRP-cage. The backbone is shown by a ribbon representation. The hydrophobic core is shown, in which the central residue Trp6 is surrounded by Tyr3, Leu7, Gly11, Pro12, Pro18, and Pro19.

Trp-cage is a designed 20-residue protein (Asn1-Leu2-Tyr3-Ile4-Gln5-Trp6-Leu7-Lys8-Asp9-Gly10-Gly11-Pro12-Ser13-Ser14-Gly15-Arg16-Pro17-Pro18-Pro19-Ser20; PDB 1L2Y.pdb) [86]. NMR spectroscopy [86] and X-ray crystallography [87] have been applied to determine the structure of this mini-protein. It contains an α-helix (Leu2-Lys9), a 3^10^-helix (Gly11-Ser14), and a polyproline II helix (Pro17-Pro19) (Figure 5). The native structure is stabilized by hydrogen bonding between the carbonyl oxygens of Arg16 and Hϵ1 of the TRP6 and the salt bridges formed between the Asp9 and Arg16 residues. Additionally, it also consists of a hydrophobic cage formed by the packing of Tyr and Trp6 residues around the Gly11, Pro12, Pro18, and Pro19 residues, which is crucial for maintaining the integrity of the structure [86,88,89].

Due to its structural simplicity and rapid folding dynamics, Trp-cage has been extensively studied both experimentally [90,91,92,93,94,95,96,97,98] and computationally [99,100,101,102,103,104,105,106,107,108,109,110,111,112,113,114] in order to understand the folding mechanisms of the protein in water. To date, the folding mechanisms of the TRP-cages still remain elusive. In some experimental [90,91,92,93] and simulation [99,100,101,102] works, these meant that the folding kinetics may have been two-state. However, from the other experimental [94,95,96,97,98] and simulation [103,104,105,106] studies, they suggested that its kinetics were not two-state. Additionally, some experimental works indicated the presence of well-defined on-pathway intermediates [94,95,96,97,98], which was in contrast to the suggestion that the folding was downhill [115,116].

To date, two characteristic folding pathways have been identified for the mini-protein [107,108,109,110,111]. In one pathway (I), the collapse of the hydrophobic core precedes the formation of the α-helix, and, in the other pathway (II), the α-helix is (partially) formed in the initial stage of folding, which is followed by the collapse of the hydrophobic core. Some MD simulations of the TRP-cage folding [103,105,111] suggest that pathway I is dominant, or even exclusive, as in experiment [95]. However, the others [102] mean that pathway II prevails, similar to what was observed experimentally [93,98]. Additionally, folding pathways may be dependent on temperatures. Based on MD simulations, pathway I may be observed at room and nearby temperatures [103,105,111], and pathway II is found at melting and higher temperatures [102].

To understand the folding mechanism of the Trp-cage in water, MD simulations were carried out. Compared to explicit solvents, the implicit solvent models led to folding rates faster than the experimental values, but the relative rates of formation of the secondary structural elements were comparable to the values observed experimentally [107]. In fact, several implicit solvent models have been reported to yield correct refolded structures of the Trp-cage [117,118]. It should be noted that the melting temperatures obtained in the simulations were typically much higher than the experimental value *T*m ≈ 315 K [86,90], both in the simulations with an explicit solvent (440 K [99] and 455 K [112]) and with an implicit solvent (400 K [113], 450 K [100], and 468 K [102]). It is noted that the specific explicit solvent’s force field combination (AMBER99SB or AMBER99SB-ILDN with TIP3P water) provides realistic accuracy in predicting the Trp-cage melting temperature [101]. In this work, the AMBER99SB-ILDN force field with a generalized Born-based implicit solvent model was used to investigate the folding mechanism of the TRP-cage in water (see Methods). In this study, the total 2000 ns MD simulations were, respectively, conducted at 315 K, 320 K, and 350 K.

Based on the “beacon” model of folding, the protein may be divided into the FK and remaining non-FK regions. For the Trp-cage, the FK is the α-helix (Leu2-Lys9), and the rest of the protein is related to the non-FK region. Hydrophobic interactions play an important role in the whole folding process, especially in the initial structural collapse. Additionally, protein folding is an enthalpic process. During folding, it is necessary to form the α-helix, which may be related to the intramolecular hydrogen bondings within the α-helix’s structure. It may be regarded as the necessary pathway during folding. In addition, this is reflected in the local free-energy basin in the FEL. Regarding the non-FK region, dynamic conformations are expected in the folding process. In the final folding stage, the mini-protein is folded into the native structure, which is due to the combined effects of various interactions, such as van der Waals interactions, salt bridges, etc. Therefore, the intermediate is expected in the folding process of the TRP-cage in water.

**Figure 6:**
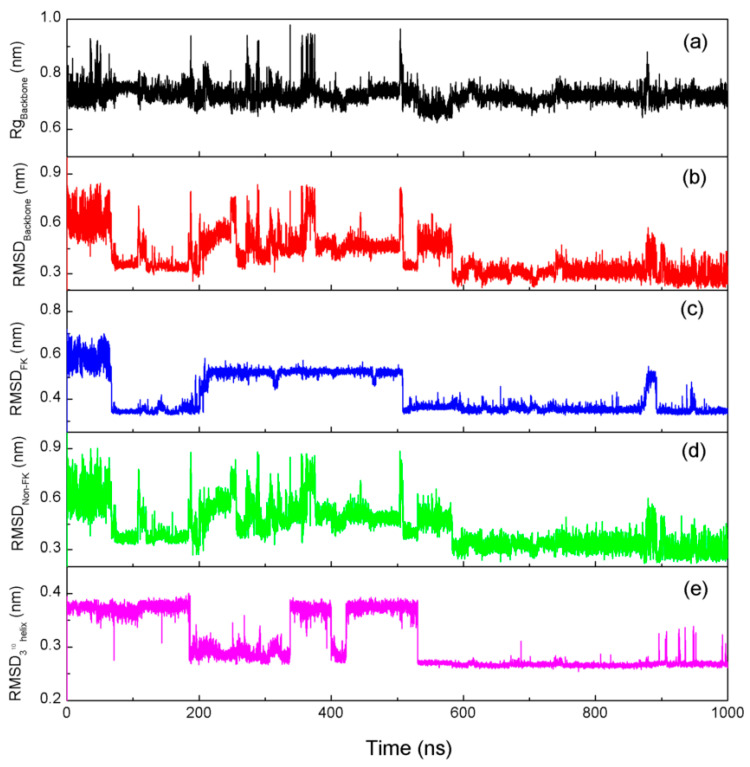
The backbone Rg (**a**) and RMSD (**b**) during folding at 315 K. To investigate the conformation changes in the folding process, the RMSD of the backbone related to the FK (α-helix) (**c**) and non-FK (**d**) regions of the Trp-cage and the 3^10^-helix (**e**) are also calculated.

To monitor the conformational change in the Trp-cage during folding at 315 K, the root-mean square deviation (RMSD) relative to the NMR structure [86] (PDB: 1L2Y) was determined (Figure 6b). Based on the simulations, the backbone RMSD decreased to 0.35 nm at about 68 ns and started fluctuating up to 0.8 nm. Once the structure fell to 0.3 nm for about 590 ns, it stayed there for the remainder of the simulation. Additionally, it was found that the lowest backbone RMSD value was 0.206 nm. Therefore, the native state was the most-stable state sampled during the simulation. To estimate the effective compactness of the Trp-cage mini-protein, the radius of gyration (Rg) of the backbone atoms of the mini-protein was also calculated. From Figure 6a, the MD trajectory visited both extended conformations (Rg ≥ 0.95 nm) and compact unfolded structures (Rg ≤ 0.75 nm) many times before it doled. The value of Rg may remain stable after the native structure is reached.

Regarding the FK region of the Trp-cage, it is related to the α-helix. In comparison to the NMR structure, the calculated RMSD of the α-helix decreases from 0.6 nm to 0.35 nm at about 70 ns, and then it increases up to 0.5 nm at 200 ns (Figure 6c). When the RMSD_FK (α-helix)_ decreases to 0.35 nm at 500 ns, it remains stable. Compared with the RMSD_FK (α-helix)_, evident changes and fluctuations of the RMSD_non-FK_ may be found for the non-FK regions of the Trp-cage before 590 ns (Figure 6d). These may be closely related to the conformational changes in the protein, as shown in RMSD_Backbone_ (Figure 6b). Additionally, evident decreases can be observed for the changes and fluctuations of the RMSD_non-FK_ after 590 ns (Figure 6d). These mean that the protein is folded into the native three-dimensional structure.

To understand the conformational changes in the ternary structure of the mini-protein, the RMSD of the 3^10^ Helix of the backbone is determined during folding at 315 K. In comparison with the NMR structure of the 3^10^ Helix, the RMSD_310 Helix_ decreases to 0.27 nm at 520 ns and keeps stable in the remaining simulation time (Figure 6e). Therefore, the formation of the 3^10^ Helix α-helix is later than the formation of the secondary structure (α-Helix) of the Trp-cage. From the MD simulations, sequential folding may be found for the Trp-cage in water.

**Figure 7:**
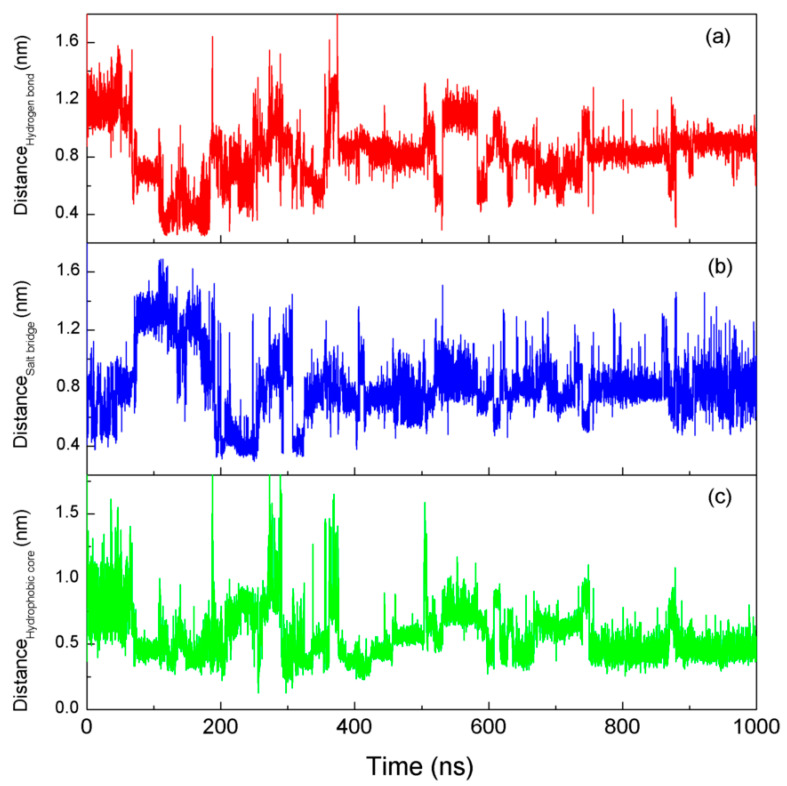
The distances of hydrogen bondings (**a**), salt bridges (**b**), and hydrophobic cores (**c**) during the simulations at 315 K.

Additionally, other factors [86,88,89] may also play a very crucial role to keep the native structure of the Trp-cage stable. These factors are (I) the formation of hydrogen bonds (Figure 7a) between the Hϵ1 of the Trp6 residue and the backbone carbonyl (C=O) of the Arg16 residue, (II) the salt bridge (Figure 7b) between the two residues Asp9 and Arg16, and (III) a hydrophobic core (Figure 7c) containing Tyr3, Gly11, Pro12, Pro18, and Pro19 residues surrounding the central residue Trp6. Based on the MD simulations, the corresponding distances for the center of mass are, respectively, determined for the hydrogen bond, the salt bridge, and hydrophobic core (Figure 7). Evident changes and fluctuations are found for these distances before 590 ns. Additionally, they become relatively stable after 590 ns. This is related to the formation of the native structures of a protein in water. Of course, it is necessary for the corresponding residues to approach before they are affected by these interactions. In fact, this is related to hydrophobic interactions, which lead to the minimization of the surface area to volume ratio to maximize the hydrogen bondings of water.

**Figure 8:**
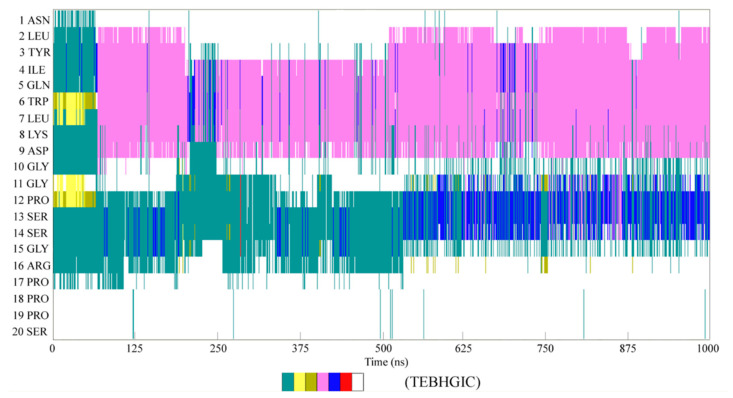
DSSP analysis of the Trp-cage during folding at 315 K.

To investigate the folding mechanisms of the Trp-cage in water, the secondary structure was analyzed by the DSSP tool in VMD [119] (Figure 8, Figures S1 and S2). It was found that the folding pathway of the Trp-cage may have been dependent on the temperature. However, it was necessary to form the α-helix during folding. This may have been related to the intramolecular hydrogen bondings within the α-Helix. During folding at 315 K, the α-helix of the mini-protein appeared at 67.5 ns and may have persisted in the remaining simulation time (Figure 8). In other words, after the hydrogen bondings within the secondary were formed, they may have been preserved. This was slightly different from the calculated RMSD of the α-Helix (Figure 6c), which meant that the secondary structure was destabilized from 200 ns to 520 ns.

Based on the above thermodynamic analysis, protein folding is an enthalpic process. Therefore, folding may be guided by the interactions between the atoms of a protein. Due to the strong intramolecular hydrogen bondings within the FK region, it is necessary to form the α-Helix before the mini-protein is folded into the native structure. In the MD simulations, three to four α-helical *i*, *i* + 4 main-chain hydrogen bonds are found during the formation of the secondary structure of the Trp-cage. Additionally, after the secondary is formed, it may be preserved in the remaining folding time, which may be regarded as the necessary pathway in the folding process. In the final folding stage, the folding process may be modulated by the hydrogen bondings between the side chains, salt bridges, and hydrophobic interactions to attain the native structure.

**Figure 9:**
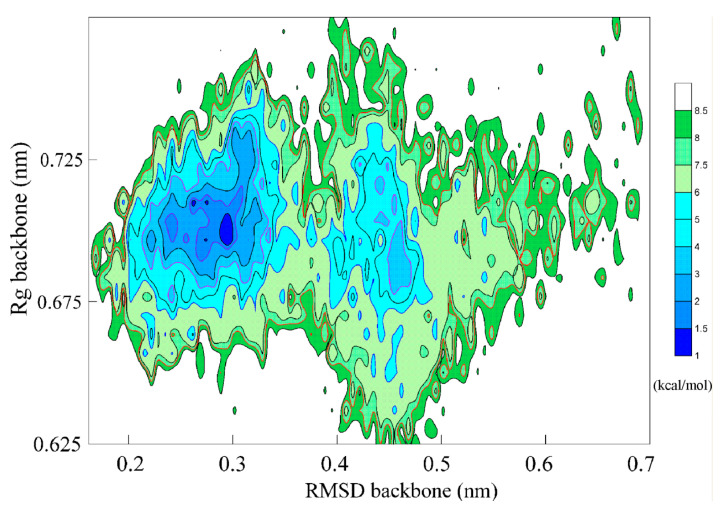
The FEL during folding of the Trp-cage at 315 K. The intermediate is found during folding, which is reflected on the local basin of the free energy.

To understand the folding mechanism of the Trp-cage in water, the FEL of the miniprotein was calculated using the g_sham package in GROMACS v4.5.2, which was expressed as

(12) $$\Delta G=-kT\ln\frac{P\left(x_{i}\right)}{P_{\max}\left(x\right)}$$

where Δ*G* was the free energy, *P*(*x_i_*) was the probability of being in state *i*, *P*_max_(*x*) was the probability of the most observed state, *k* was the Boltzmann constant, and *T* was the temperature (315 K). Based on the simulations, the FEL may have been determined as a function of the backbone RMSD and Rg (Figure 9). During the Trp-cage folding from a linear chain to a native structure, there was a decrease in free energy. During folding, three local Gibbs energy basins are found in the FEL. Different from the classical picture with only two states, the intermediate state is found during folding, in which the specific conformation (α-Helix) is formed in the FK region, and dynamic conformation is found for the non-FK region. In fact, the presence of a metastable intermediate state has been observed in many computational and experimental works [94,95,96,97,98].

**Figure 10:**
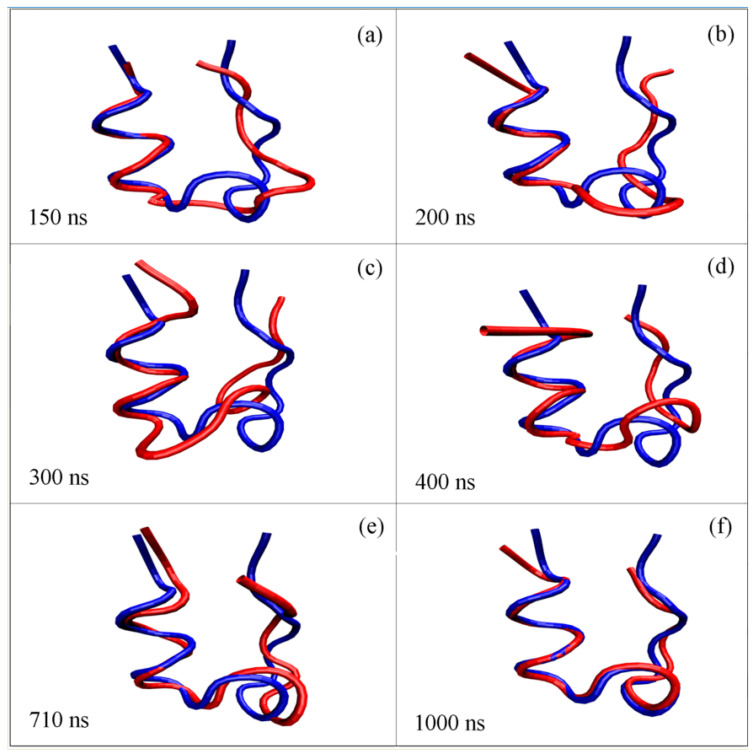
The conformational changes of Trp-cage during folding at 315 K. The NMR structure (1L2Y.pdb) of the Trp-cage is drawn in blue. The conformations of the mini-protein at various times are shown in (**a**–**f**), which are shown in red. Different from the specific conformation (α-Helix) of the FK region, dynamic conformations are found for the rest of the Trp-cage (non-FK region).

From the MD simulations, the intermediate was found during folding, which was related to the various conformations of the FK and non-FK regions. From the “beacon” structural model, the specific conformation was expected for the FK region during folding, which was related to the intramolecular hydrogen bondings within the region. Additionally, after the α-Helix was formed, it was preserved in the following simulation time (Figure 10). However, different from the FK regions, dynamic conformation may have been expected for the non-FK regions of the miniprotein. This may have been reflected on the conformational changes and fluctuations during folding (Figure 10a–d). After the RMSD was equilibrated at 590 ns, the representative conformation was shown in Figure 10e. This was obtained through cluster analysis, which was conducted through the g_cluster tool of GROMACS. Additionally, the final conformation was also shown (Figure 10f). Additionally, with decreasing the free energy during folding, especially as the α-Helix was formed, this led to the decrease in the conformal changes in non-FK regions until the native structure was reached (Figure 6d).

Two folding pathways have been identified for the Trp-cage in water, which may have been dependent on temperature. This was mainly related to the Gibbs energies of the hydrophobic interactions and the α-helixes of the mini-protein. Hydrophobic interactions may be involved in the whole folding process of the TRP-cage in water. From Equation (7), the strength of the hydrophobic interactions may have been related to *n_Interfacial→bulk water_* and Δ*G_DDAA_*. In the folding process, *n_Interfacial→bulk water_* was related to the ratio of the surface area to volume of the folded Trp-cage. From Raman spectroscopic studies [45,46], the increase in temperature may have led to the decrease in Δ*G_DDAA_*. With increasing temperature, more interfacial water molecules (*n_Interfacial__→bulk water_*) were necessarily transformed into bulk water so that the hydrophobic interactions may have been equivalent to the Gibbs energies of the α-helixes. Of course, this was accompanied with the smaller ratio of surface area to volume of the proteins of the TRP-cage, which was related to the structural collapse of the mini-protein in water. Further study may be necessary.

**Figure 11:**
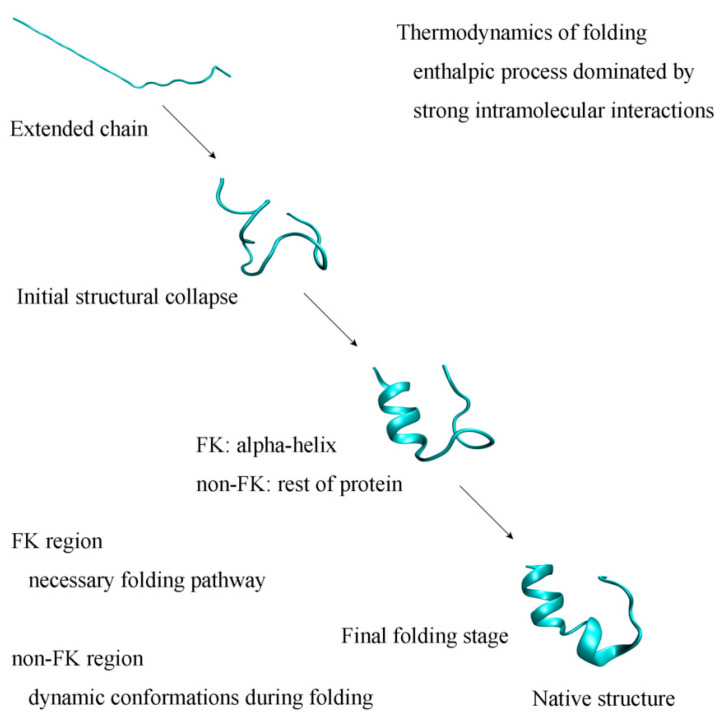
The folding mechanism of the TRP-cage in water.

In this work, the total 2000 ns MD simulations were, respectively, carried out at 315 K, 320 K, and 350 K. Based on the simulations, it was necessary to form the α-helix during folding, which may have been regarded as the necessary pathway. Of course, this was related to the intramolecular hydrogen bondings within the secondary structures of the the TRP-cage. From the work, the intermediate state may have been found during the folding of the Trp-cage from an extended chain to a three-dimensional structure. It was expressed as initial state→intermediate state→final structure (Figure 11). This may have been reflected in the local basins of the Gibbs-free energy on the FEL of the TRP-cage in water. Additionally, this was also in accordance with the “beacon” structural model discussed above.

In thermodynamics, folding is an enthalpic process that is due to various intermolecular interactions, especially hydrophobic interactions and intramolecular hydrogen bondings in proteins. Owing to hydrophobic interactions, folding leads to the structural collapse from a linear structure to a three-dimensional structure. With decreasing the separations between the atoms of proteins, these increase the direct interactions within proteins, which may affect the folding process. Therefore, hydrophobic interactions play an important role in the whole folding process, especially in the initial folding stage. In other words, protein folding may be driven by hydrophobic interactions. Based on the work, protein is divided into the FK regions related to the necessary pathways and the remaining non-FK regions. Due to the formations of intramolecular hydrogen bondings within proteins, specific conformations may be expected for the FK regions, such as α-helixes and β-sheets. Proteins may contain several FK regions that are expected to sequentially form during folding. Therefore, they may be regarded as the “beacons” of protein folding. Additionally, dynamic conformations are expected for non-FK regions in the folding process. In the final folding stage, a native structure is obtained through the combined effects of various interactions, such as hydrophobic interactions, salt bridges, van der Waals interactions, hydrogen bondings related to side chains, etc.

## 5. Methods

MD simulations could provide insight into the folding pathways with unprecedented spatial and temporal resolutions. Therefore, they are widely utilized to investigate the folding mechanisms of proteins in water. In this study, to investigate the folding mechanism of the TRP-cage in water, MD simulations were carried out through the GROMACS 4.5.2 [120,121] package.

In this work, the AMBER99SB-ILDN force field [122] was utilized to describe the interatomic interactions. The water molecules were simulated using the generalized Born (GB) solvation model. This was used without PBC (periodic boundary conditions) and no pressure coupling. Velocity rescaling (v-rescale) thermostat dynamics were used to control the temperatures. Additionally, the LINCS algorithm [123] was used to constrain the covalent bonds involving the hydrogen atoms. Salt concentrations were 0.2 M. The Trp-cage was, first, energy minimized for 50,000 steps using the steepest descent algorithm. Then, the total 2000 ns MD simulations were, respectively, carried out at 315 K, 320 K, and 350 K. A time step of 2 fs was used.

## 6. Conclusions

In combination with our recent studies on hydrophobic interactions, this work is devoted to investigating the folding mechanisms of proteins in water. From this work, the following conclusions were derived:

(1) Hydrophobic interactions are regarded as the fundamental driving forces in the folding process, especially in the initial stage. Due to hydrophobicity, this leads to the structural collapse of a protein in water. By decreasing the distances between the residues of proteins, the direct interactions between them become important, which may affect the folding process.

(2) Protein folding is a process of enthalpy to overcome the loss of conformational entropy arising from folding. In fact, various interactions may be involved in protein folding. Based on thermodynamic analysis, folding is reasonably guided by the strong interactions within proteins, such as intramolecular hydrogen bondings related to the α-helixes and β-sheets of secondary structures.

(3) Proteins are divided into FK regions related to intramolecular hydrogen bondings and non-FK (the rest of protein) regions. During folding, specific and dynamic conformations are, respectively, expected for the FK and non-FK regions. Different from the non-FK regions, it is necessary for the FK regions to form the specific conformations in the folding process, which are regarded as the necessary pathways (or “beacons”) during folding. Additionally, sequential folding is expected for the FK regions, and an intermediate state is found during folding. In addition, they are reflected on the local basins in the FEL of folding.

## Data Availability

Not applicable.

## Supplementary Materials

The following supporting information can be downloaded at: https://www.mdpi.com/article/10.3390/molecules28135164/s1, To study the folding pathways of the TRP-cage in water, total 2000 ns MD simulations were, respectively, carried out at 315 K, 320 K, and 350 K. To investigate the folding mechanisms of the TRP-cage in water, the secondary structures were analyzed by DSSP tool in VMD [119]. They were, respectively, drawn in Figure 8, Figures S1 and S2. The trajectory files and Figures S1 and S2 are included in supplementary data. Figure S1. DSSP analysis of Trp-cage during the folding at 320 K. Figure S2. DSSP analysis of Trp-cage during the folding at 350 K.

## Sample Availability

Samples of the compounds are available from the authors.
