# Supplementary material for: The “Beacon” Structural Model of Protein Folding: Application for Trp-Cage in Water
Source: Molecules. 2023 Jul 2;28(13):5164. doi: 10.3390/molecules28135164 (PMC10343236; doi:10.3390/molecules28135164)
Supplement: Supplementary file 1 [file molecules-28-05164-s001.zip › Supplementary-Figures.pdf]

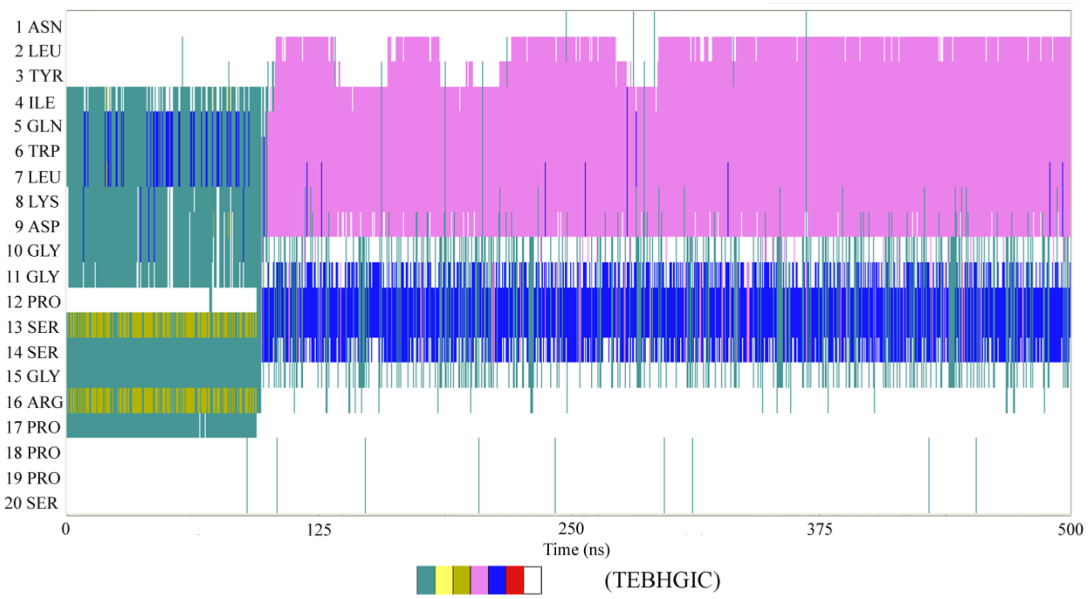

Figure s1. DSSP analysis of Trp-cage during the folding at 320 K.

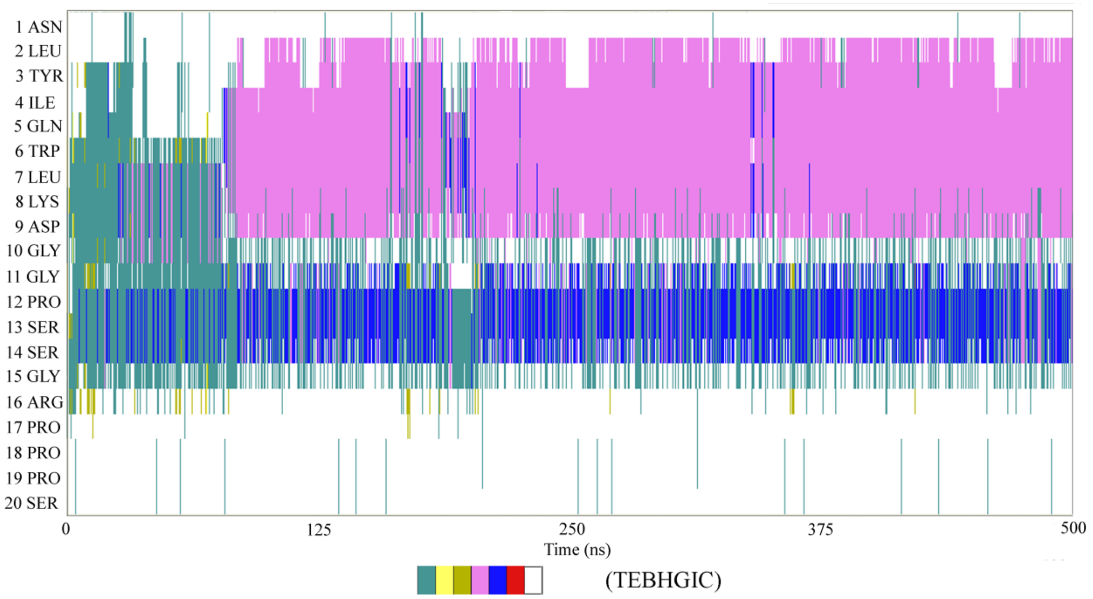

Figure s2. DSSP analysis of Trp-cage during the folding at 350 K.
